# Phospholipase Cζ (PLCζ) versus postacrosomal sheath WW domain‐binding protein (PAWP): Which molecule will survive as a sperm factor?

**DOI:** 10.1111/asj.13345

**Published:** 2020-02-10

**Authors:** Michiko Nakai, Junya Ito, Ayumi Suyama, Atsuko Kageyama, Yasuko Tobari, Naomi Kashiwazaki

**Affiliations:** ^1^ Institute of Agrobiological Sciences National Agriculture and Food Research Organization Tsukuba Japan; ^2^ Laboratory of Animal Reproduction School of Veterinary Medicine Azabu University Sagamihara Japan; ^3^ Graduate School of Veterinary Sciences Azabu University Sagamihara Japan; ^4^ Laboratory of Animal Genetics and Breeding School of Veterinary Medicine Azabu University Sagamihara Japan

**Keywords:** calcium, fertilization, oocyte, phospholipase C, sperm

## Abstract

During mammalian fertilization, sperm is fused with the oocyte's membrane, triggering the resumption of meiosis from the metaphase II arrest, the extrusion of the second polar body, and the exocytosis of cortical granules; these events are collectively called 'oocyte activation.' In all species studied to date, the transient rise in the cytosolic level of calcium (in particular, the repeated calcium increases called 'calcium oscillations' in mammals) is required for these events. Researchers have focused on identifying the factor(s) that can induce calcium oscillations during fertilization. Sperm‐specific phospholipase C, i.e., PLC zeta (PLCζ), is a strong candidate of the factor(s), and several research groups using different species obtained evidence that PLCζ is a sperm factor that can induce calcium oscillations during fertilization. However, postacrosomal sheath Tryptophan‐Tryptophan (WW)—domain‐binding protein (PAWP) was recently shown to have a pivotal role in inducing calcium oscillations in some species. In this review, we focus on PLCζ and PAWP as sperm factors, and we discuss this controversy: Which of these two molecules survives as a sperm factor?

## 1 SPERM FACTOR THEORY

Ovulated mammalian oocytes remain arrested until fertilization at the metaphase II (Ducibella, Schultz, & Ozil, 2006; Jones, 2005). In mammals, there are transient and repetitive increases in the cytosolic level of free calcium (in a phenomenon that is also known as calcium oscillations); these oscillations are induced immediately after sperm‐oocyte fusion (Kline & Kline, 1992; Miyazaki, Yuzaki, et al., 1992; Miyazaki et al., 1993). These calcium oscillations trigger the events of oocyte activation including the resumption of second meiosis, maternal mRNA recruitment, pronucleus (PN) formation, and embryonic development (Xu, Kopf, & Schultz, 1994). The pattern of rise such as amplitude and number in the cytosolic level of calcium might affect gene expressions at later stages of preimplantation embryo development and even after postnatal development (Ozil, Banrezes, Toth, Pan, & Schultz, 2006).

The mechanisms by which sperm induce calcium oscillations have been investigated in several species. In the hamster (Miyazaki, Shirakawa, et al., 1992) and mouse (Xu et al., 1994) as well as echinoderms (Carroll et al., 1997) and frogs (Nuccitelli, Yim, & Smart, 1993), the sperm‐induced calcium increase is generated by a release of calcium from 1,4,5‐inositol trisphosphate (IP_3_)‐sensitive intracellular calcium stores. Since IP_3_ is generated by a phospholipase C (PLC)‐mediated hydrolysis of phosphatidyl inositol 4,5‐bisphosphate (PI(4,5)P_2_), the activation of PLC and the production of IP_3_ are required for the induction of calcium oscillations. The details of the mechanisms underlying the calcium release at fertilization in vertebrates have not yet been established — especially the interaction between the sperm and oocyte in this process.

**Figure 1:**
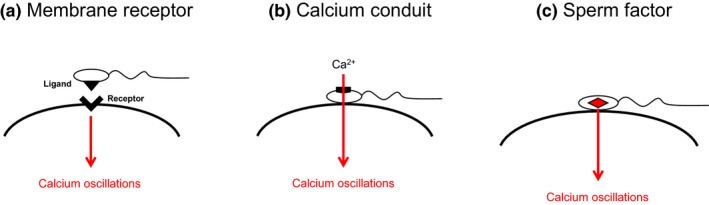
Hypotheses regarding the sperm‐inducing calcium release during fertilization in mammals

Three major hypotheses have been proposed to date to explain how sperm induce calcium oscillations during mammalian fertilization (Ito, Parrington, & Fissore, 2011; Kurokawa, Sato, & Fissore, 2004; Runft, Jaffe, & Mehlmann, 2002; Swann, Saunders, Rogers, & Lai, 2006; Whitaker, 2006). The first hypothesis is known as the 'membrane receptor hypothesis' and contends that an oocyte surface receptor is engaged by a sperm ligand, triggering the production of a signaling pathway that triggers calcium release via the PLC of the oocyte (reviewed by Swann & Parrington, 1999) (Figure 1a). The 'conduit hypothesis' proposes that extracellular calcium flows into the oocyte via the sperm during gamete fusion (Figure 1b). Both the membrane receptor hypothesis and the conduit hypothesis rely on a sperm‐oocyte interaction to induce calcium responses, and the implausibility of these hypotheses was later revealed by the success of intracytoplasmic sperm injection (ICSI), which can bypass the sperm‐oocyte interaction in the plasma membrane and is capable of inducing fertilization‐like calcium responses.

A third theory, the 'sperm factor hypothesis,' could be considered a very likely mechanism for the induction of calcium oscillations (Figure 1c). The sperm factor hypothesis suggests that immediately after sperm‐oocyte fusion, the sperm release a soluble activating factor into the oocyte (Stricker, 1999; Swann, 1996). In support of this hypothesis, the injection of sperm cytosolic extracts into oocytes resulted in calcium oscillations similar to those observed during fertilization in many species, including sea urchin (Dale & Santella, 1985), an ascidian (McDougall, Levasseur, O'Sullivan, & Jones, 2000), chicken (Kim & Gye, 2003), quail (Mizushima et al., 2009), hamster (Swann, 1990), mouse (Parrington, Swann, Shevchenko, Sesay, & Lai, 1996), rat (Ito, Shikano, Oda, et al., 2008), cow (Wu, He, & Fissore, 1997), pig (Machaty, Bonk, Kuhholzer, & Prather, 2000), human (Wilding, Kyozuka, Russo, Tosti, & Dale, 1997), horse (Hinrichs, Choi, Love, Chung, & Varner, 2006), and rabbit (Stice & Robl, 1990). The sperm factor hypothesis is now strongly supported; it appears that one or more sperm factors exist on sperm, and the factor(s) are a trigger to induce calcium oscillations and oocyte activation — at least in mammalian species.

## 2 THE DISCOVERY OF PLCζ

The calcium release component(s) of sperm cytosolic extracts has been investigated. A 33‐kDa protein isolated from a sperm extract fraction that localizes in the equator of sperm and has the ability to activate oocytes: oscillin (Parrington et al., 1996), but a microinjection of recombinant oscillin does not induce calcium oscillations (Wolosker et al., 1998). Moreover, when sperm extracts were fractionated, oscillin was not detected in the fraction with strong calcium^‐^releasing activity (Wu et al., 1997). A truncated c‐kit tyrosine kinase (tr‐kit) was also considered as a candidate sperm factor (Sette et al., 1997), and although a microinjection of tr‐kit mRNA into mouse oocytes caused complete oocyte activation, it is not yet clear whether or not tr‐kit has calcium releasing activity (Sette et al., 1997).

Saunders et al. (2002) proposed that phospholipase Cζ (PLCζ) might be the sperm‐borne oocyte‐activating factor that can induce calcium oscillations. A microinjection of RNA encoding PLCζ (Saunders et al., 2002) or recombinant PLCζ protein (Kouchi et al., 2004) induced calcium oscillations. In addition, the calcium oscillation‐inducing activity of sperm extracts was lost when the sperm extracts were pretreated with an antibody against PLCζ (Saunders et al., 2002). The level of PLCζ that is able to produce fertilization‐like calcium oscillations in a single oocyte is in the same range as the single‐sperm content of PLCζ (Saunders et al., 2002). Moreover, reducing the level of sperm PLCζ protein by transgenic RNA interference resulted in the perturbation of the patterns of calcium oscillations after fertilization (Knott, Kurokawa, Fissore, Schultz, & Williams, 2005).

To date, PLCζ has been detected in many mammalian and nonmammalian species (Table 1), including mouse (Ito, Shikano, Kuroda, et al., 2008; Saunders et al., 2002), rat (Ito, Shikano, Oda, et al., 2008), human (Cox et al., 2002; Ito, Shikano, Oda, et al., 2008), cynomolgus monkey (Cox et al., 2002), cow (Cooney et al., 2010; Malcuit et al., 2005), pig (Kurokawa et al., 2005; Yoneda et al., 2006), horse (Bedford‐Guaus et al., 2011; Sato et al., 2013), medaka fish (Coward et al., 2011), and quail (Mizushima et al., 2014). PLCζ orthologues were identified even in pufferfish species *Takifugu rubripes* (fugu) and *Tetraodon nigroviridis* (tetraodon) (Coward et al., 2011). The injection of pufferfish PLCζ crab into mouse oocytes failed to trigger calcium oscillations, unlike other animal species PLCζ. Interestingly, PLCζ is not expressed in pufferfish testes but is expressed in the ovaries, suggesting that its mechanism of action and physiological role at fertilization may differ from vertebrate species.

**Table 1:** Mammalian species studied for induction of oocyte activation by PLCζ or PAWP

Species	PLCζ	PAWP^a^
Mouse	Saunders et al. (2002)	Wu et al. (2007)
	Yoda et al. (2004)	
	Fujimoto et al. (2004)	
	Coward et al. (2006)	
	Kurokawa et al. (2007)	
	Ross et al. (2008)	
	Bedford‐Guaus, Yoon, Fissore, Choi, and Hinrichs (2008)	
	Sato et al. (2013)	
Human	Cox et al. (2002)	Wu et al. (2007)
	Yu, Saunders, Lai, and Swann (2008)	
	Ito, Shikano, Oda, et al. (2008)	
	Yoon et al. (2012)	
	Sato et al. (2013)	
Rat	Ito, Shikano, Oda, et al. (2008)	
	Sato et al. (2013)	
Cow	Cooney et al. (2010)	Wu et al. (2007)
	Sato et al. (2013)	
Horse	Bedford‐Guaus et al. (2011)	
	Sato et al. (2013)	
Pig	Yoneda et al. (2006)	
	Nakai et al. (unpubl.)	
Monkey	Cox et al. (2002)	

a Successful molecular cloning was reported, but microinjection of protein or cRNA was not examined.

## 3 THE STRUCTURE AND FUNCTION OF PLCζ

**Figure 2:**
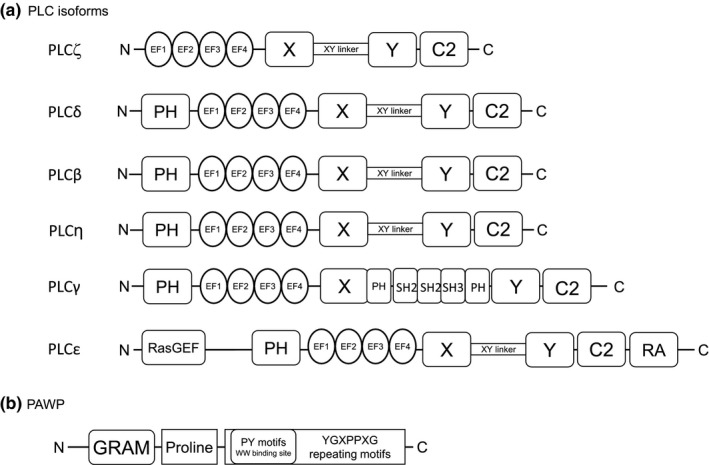
Structural features of PLC isoforms and PAWP. PLCζ is composed of four EF‐hand (EF1‐EF4) domains in the N terminus, catalytic X and Y domains, XY linker region, and a C2 domain in the C terminus. PLCζ is smaller than other PLC isoforms (δ, β, η, γ, ε). (B) The N‐terminus of PAWP exhibits sequence similarity with WW domain‐binding protein 2. In the proline‐rich C‐terminus, PAWP contains an unidentified repeating motif (YG*X*PP*X*G where *X* represents any residue) and a PY motifs, which is always found overlapping with the YG*XPPXG* motif

**Figure 3:**
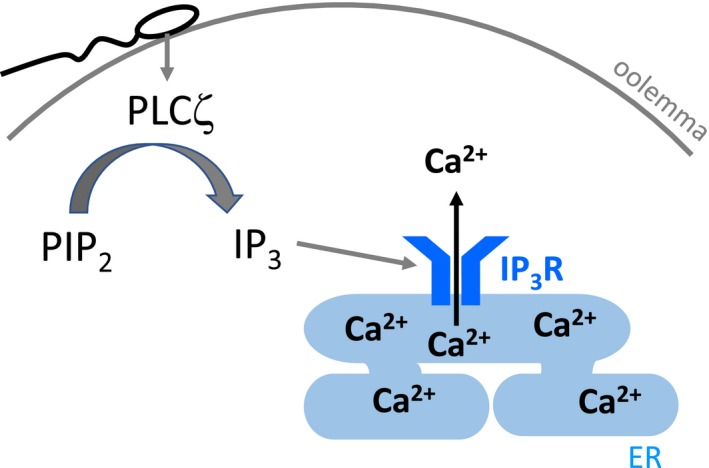
Mechanism of a rise of intracellular calcium level via PLCζ. After the sperm‐oocyte fusion, PLCζ is released from sperm into ooplasm. The PLCζ produces inositol trisphosphate (IP3) by hydrolyzing phosphatidylinositol 4,5‐bisphosphate (PI(4,5)P2) and leads to release of calcium from the endoplasmic reticulum (ER) via IP3‐receptor (IP3R)

There are 13 known mammalian PLC isozymes, and they are classified according to their structure into six types: PLC‐beta (PLCβ 1–4), ‐gamma (PLCγ 1, 2), ‐delta (PLCδ 1, 3, 4), ‐epsilon (PLCε), ‐ zeta (PLCζ), and ‐eta (PLCη 1, 2) (Fukami, Inanobe, Kanemaru, & Nakamura, 2010; Kadamur & Ross, 2013; Suh et al., 2008), of which PLCζ is the smallest in molecular size (Rebecchi & Pentyala, 2000). PLCζ is characterized by its structure: it is composed of four EF‐hand (EF1–EF4) domains in the N terminus, catalytic X and Y domains, and a C2 domain in the C terminus that is common to other types of PLC (Figure 2a). However, PLCζ lacks the N‐terminal PH domain that is found in PLCβ, ‐γ, ‐δ, and ‐ε, and the N‐terminal PH domain is the site for the interaction of PLCβ, ‐γ, ‐δ, and ‐ε with membrane phospholipids (Katan, 1998). PLCζ produces IP_3_, which binds to IP_3_ receptor on the endoplasmic reticulum and induces the release of calcium via the hydrolysis of membrane PI(4,5)P_2_ (Figure 3). It is thus not yet known how PLCζ, which lacks the PH domain, binds to phosphoinositides.

PLCζ has two functional properties: the induction of high calcium oscillations, and high calcium sensitivity in the PI(4,5)P_2_ hydrolyzing activity. PLCζ and PLCδ1, which exhibits comparatively high homology with PLCζ, have similar affinity for PI(4,5)P_2_, but PLCζ is extremely sensitive to calcium compared to PLCδ1 (Nomikos et al., 2005). These properties may depend on the N‐terminal EF‐hand domains. The absence of all of the EF hands in PLCζ resulted in a complete loss of both the PI(4,5)P_2_ hydrolyzing activity and the calcium oscillation‐inducing activity (Kouchi et al., 2005).

EF1 and EF2 are important for the PLCζ activity, as the deletion of EF1 and EF2 (PLCζ∆EF1‐2) remarkably reduced the PLCζ activity (Kouchi et al., 2005). However, a point mutation in EF1 or EF2 did not cause a marked decline in the calcium sensitivity of the PI(4,5)P_2_‐hydrolyzing activity (Kouchi et al., 2005). It has thus been suggested that the two EF‐hand domains (rather than a calcium‐binding site) play a structural role in the activation of the catalytic activity (Kouchi et al., 2005). On the other hand, the short‐PLCζ (s‐PLCζ), which lacks the three EF‐hand domains EF1, EF2, and EF3, has much less calcium oscillation‐inducing activity than PLCζ∆EF1‐2 (Kouchi et al., 2005), and thus the EF3 domain is considered responsible for the high calcium sensitivity of the PI(4,5)P_2_‐hydrolyzing activity of PLCζ, and the EF3 domain is thought to have the important role of the induction of calcium oscillations (Kuroda et al., 2006). The enzymatic activity of PLCζ seems to require a specific matching in the PLCζ molecule between the four EF‐hand domains and the other region, because the replacement of PLCζ in the region from the X domain to the C terminus with PLCδ1 resulted in no detectable PLC activity even when the four EF‐hand domains were normally prepared (Kouchi et al., 2005).

The C2 domain of PLCζ, including the 37 external amino acids in the C terminal, is a prerequisite for PLC activity and the induction of calcium oscillations. The deletion of the C2 domain (PLCζ∆C2) resulted in a complete loss of PLC activity (Kouchi et al., 2005). Moreover, the PLCζ∆C2 was unable to trigger the calcium rise (Kouchi et al., 2005; Nomikos et al., 2005). The C2 domain has been characterized as a membrane‐associated and intermolecular interaction domain in a variety of proteins (Medkova & Cho, 1999). The activation mechanism of PLCζ as well as that of PLCδ is unknown, because they lack a regulatory domain such as the G protein binding site of PLCβ or the SH domain of PLCγ for phosphorylation by tyrosine kinase. The C2 domain has been suggested to take part in the calcium‐dependent subcellular membrane targeting by several lipid‐metabolizing enzymes (Bittova, Sumandea, & Cho, 1999; Essen, Perisic, Cheung, Katan, & Williams, 1996; Grobler & Hurley, 1996; Lomasney et al., 1999; Zheng, Krishnamoorthi, Zolkiewski, & Wang, 2000). It is thus possible that the C2 domain of PLCζ also functions as the PI(4,5)P_2_ binding site. However, it has been also reported that the C2 domain does not play a direct role in the interaction with PI(4,5)P_2_, because PLCζ∆C2 showed binding to PI(4,5)P_2_ that was equivalent to that of wild‐type PLCζ (Nomikos et al., 2011), and the PLCζ X‐Y linker region, not the C2 domain, showed strong binding to PI(4,5)P_2_ (Nomikos et al., 2011).

The C2 domain of PLCζ has an affinity to PI(3)P or PI(5)P irrespective of changes in calcium (Kouchi et al., 2005; Nomikos et al., 2011). The association of the C2 domain with PI(3)P or PI(5)P may cause an inhibitory regulation of PLC activity, because the presence of PI(3)P or PI(5)P reduced the PI(4,5)P_2_ hydrolytic activity of PLCζ (Kouchi et al., 2005). The meaning of the inhibitory effects of PI(3)P or PI(5)P on PLCζ has not been clear, but it seems that there is a regulatory mechanism of the enzymatic activity of PLCζ via the interaction between the C2 domain and phospholipids.

The X and Y catalytic domains are associated with the catalytic activity common to all phosphoinositide‐specific PLCs. The X and Y catalytic domains were reported to be capable of binding and hydrolyzing PI(4,5)P_2_, because even the X and Y catalytic domain alone, which is deleted from the C2 and EF hand domains of PLCζ, exhibits well over half of the activity of the full‐length PLCζ (Nomikos et al., 2005). In addition, the point mutation of Asp^210^ (which corresponds to a calcium‐binding site responsible for the enzymatic activity of PLCδ1) to arginine in the X catalytic domain of PLCζ caused the loss of calcium oscillation‐inducing activity (Kuroda et al., 2006). The X catalytic domain could thus affect the calcium oscillation‐inducing activity.

One of the functional properties of PLCζ is its nuclear translocation ability. The nuclear localization signal sequence, which is thought to be a binding site of the nuclear transport receptor, is at amino acids 374–381 in the X‐Y linker region (Kuroda et al., 2006; Nakanishi et al., 2008). The putative nuclear localization signal (NLS) sequence Lys^374^‐Ala^383^ fused with a fluorescent protein, accumulated into both the nucleoplasm and nucleoli in COS‐7 cells (Kuroda et al., 2006). In addition to the X‐Y linker region, the region including Trp^13^, Phe^14^, and Val^18^ in the N terminus of the EF1 domain is also essential for the translocation (Kuroda et al., 2006; Yoda et al., 2004). The s‐PLCζ lacking the three EF‐hand domains (∆EF1‐3) is unable to assemble in the pronucleus (Yoda et al., 2004). However, PLCζ∆EF2‐3 or ∆EF2‐4 loses the translocation ability even when the EF1 domain is present (Kuroda et al., 2006). Therefore, all EF‐hand domains, not only the EF1 domain, seem to play a role in the translocation of PLCζ.

The PLCζ or sperm factor accumulates in the PN (Kono, Carroll, Swann, & Whittingham, 1995; Larman, Saunders, Carroll, Lai, & Swann, 2004; Ogonuki et al., 2001; Sone et al., 2005; Yoda et al., 2004), and the calcium oscillations in fertilized mouse oocytes cease at about the time of PN formation (Deguchi, Shirakawa, Oda, Mohri, & Miyazaki, 2000; Jones, Carroll, Merriman, Whittingham, & Kono, 1995). It has thus been suggested that the translocation of PLCζ plays a key role in the cessation of calcium oscillations at the interphase of the cell cycle (Kono et al., 1995; Larman et al., 2004; Marangos, FitzHarris, & Carroll, 2003). However, it has been reported that the PLCζ from human, rat, medaka fish (Ito, Shikano, Oda, et al., 2008), horse (Sato et al., 2013), and bovine (Cooney et al., 2010) is unable to accumulate in the PN. Calcium oscillations did not terminate at the time point of PN formation in mouse oocytes injected with pig PLCζ cRNA (Yoneda & Watanabe, 2015). The s‐PLCζ, which loses the nuclear translocation ability and localizes in the cytoplasm after oocyte activation, had no negative effects on the pronucleus formation and subsequent development in mouse (Nakanishi et al., 2008). The physiological function of PLCζ translocation to the PN remains uncertain.

## 4 THE DIFFERENCES IN PLCζ AMONG SPECIES

PLCζ mRNA is expressed in spermatocytes in hamster (Young, Grasa, Coward, Davis, & Parrington, 2009) and in elongated spermatids in mouse, pig, and quail (Mizushima et al., 2009; Yoneda et al., 2006), but it seems that the spermatogenic cells do not acquire the oocyte activation ability and the calcium oscillation‐inducing ability at the same time even when PLCζ mRNA is expressed. In mouse and rat, elongated spermatids induce both oocyte activation (which involves the extrusion of the second polar body and the formation of more than one PN) and a transient rise of the intracellular calcium concentration, but not an oscillation pattern (Yazawa, Yanagida, Katayose, Hayashi, & Sato, 2000). Round spermatids are unable to induce either of these events in the above‐cited species. Round spermatids in hamster, rabbit, and human can induce oocyte activation and an increase in the intracellular calcium concentration, in a transient pattern (Yazawa et al., 2000). The cells grow along with spermatogenesis, and the pattern of the increase in the intracellular calcium concentration changes from a transient pattern to an oscillation pattern. Although in hamster and rabbit the calcium oscillations are triggered by elongated spermatids, spermatozoa induce the oscillations in mouse and rat. In human, round spermatids have shown calcium oscillation‐inducing ability (Yazawa et al., 2000). The timing of the functional maturation of spermatogenic cells differs among species.

PLCζ has been detected in the postacrosomal region (Yoon & Fissore, 2007) or acrosomal and postacrosomal regions (Young et al., 2009) of mouse sperm, in the acrosomal region of rat sperm (Seita, Ito, & Kashiwazaki, 2009), in the equatorial (Yoon et al., 2008) or equatorial and postacrosomal regions (Heytens et al., 2009) of human sperm, in the equatorial region of bull sperm (Yoon & Fissore, 2007), in the acrosomal and postacrosomal regions of hamster sperm (Young et al., 2009), in the acrosome, equatorial region, head‐midpiece junction and tail of equine sperm (Bedford‐Guaus et al., 2011), and in the postacrosomal region and tail of pig sperm (Nakai et al., 2011). The PLCζ distribution patterns seem to be affected by sperm capacitation and the acrosome reaction. After capacitation or acrosome reactionin human, the proportion of sperm displaying the equatorial/acrosomal location of PLCζ decreased, whereas sperm with the equatorial/postacrosomal pattern of PLCζ localization increased (Grasa, Coward, Young, & Parrington, 2008). It is not clear that PLCζ protein actually moves to the postacrosomal region, and it is not yet known whether the accessibility of the PLCζ to an antibody changes during the capacitation and acrosome reaction.

Considering the possibility that a sperm factor is released into the oocyte cytoplasm even before the sperm's full entry, it can be speculated that a sperm factor would reside around the equatorial region, which is the site at which the sperm‐oocyte fusion takes place during fertilization (Yanagimachi, 1994). However, in some species PLCζ is localized at the acrosomal and/or tail regions. In mouse and hamster, the acrosomal/postacrosomal localization in uncapacitated sperm changes to postacrosomal localization after the capacitation and acrosome reaction (Young et al., 2009). The disappearance of acrosomal localization suggests that the acrosomal PLCζ population might play a role in the acrosome reaction (Young et al., 2009). It would be interest to determine whether PLCζ has functions other than the induction of oocyte activation.

## 5 DISCOVERY OF PAWP

After the discovery of PLCζ, evidence from many studies strongly indicated that PLCζ is the functional sperm factor, at least in mammalian species. It is not yet known whether PLCζ is the sole sperm factor and whether it is indispensable for oocyte activation during fertilization. In 2007, Dr. Richard Oko and his colleagues described a novel alkaline extractable protein of the sperm head that resides exclusively in the postacrosomal sheath region of the perinuclear theca (PT) (Wu et al., 2007). The protein shares the sequence homology with the N‐terminal half of WW domain‐binding protein 2 (WBP2), and the C‐terminal half of the protein is unique and rich in proline. The protein was named PAWP (postacrosomal sheath WW domain‐binding protein), and is also known as WBP2NL.

With the use of an antibody, PAWP was detected in the cytoplasmic lobe of spermatids beginning to undergo elongation in mouse, rabbit, pig, cow, rhesus monkey, and human (Aarabi et al., 2014a, 2014b; Wu et al., 2007). The microinjection of recombinant human PAWP or alkaline PT extract into metaphase II‐arrested porcine, cow, macaque, and *Xenopus* oocytes was examined in these studies, and after microinjection, the oocytes formed pronuclei at a high rate, suggesting that PAWP could be a candidate sperm factor for oocyte activation. The co‐injection of PAWP and the PAWP‐derived competitive to Pro‐Pro‐*X*‐Tyr (PY) motif (where *X* represents any residue) suppressed the induction of pronuclear formation. Since a co‐injection of PAWP with the PY motif point‐mutated peptide can also induce pronuclear formation, one residue of PAWP is important for oocyte activation. The importance of one residue is same with PLCζ because the calcium induced activity of PLCζ was defective when Asp^210^ in the X catalytic domain was mutated (Kuroda et al., 2006). Moreover, the ICSI of porcine oocytes combined with a co‐injection of the competitive PY peptide or an anti‐recombinant PAWP antiserum prevented pronuclear formation and arrested fertilization (Aarabi et al., 2014a, 2014b). These findings supported the hypothesis that PAWP has a crucial role as a sperm factor.

The next concern was whether PAWP can induce one or more increases in the level of calcium during oocyte activation, because such an ability is one of the most important factors for a candidate sperm factor. Aarabi, Qin, Xu, Mewburn, and Oko (2010) demonstrated that in Xenopus oocytes, an injection of recombinant human PAWP causes an intracellular calcium release. Their later study confirmed that an injection of human PAWP protein triggers calcium oscillations and pronuclear formation even in mammalian oocytes (mouse and human) (Aarabi et al., 2014b). In that study, the authors also showed that the sperm‐induced calcium oscillations can be blocked by a co‐injection of a competitive PY peptide. It suggests that both sperm‐derived PAWP and an oocyte‐derived group‐I WW domain (WWI domain)‐containing protein, such as “Yes‐Associated Protein (YAP)”, “neural precursor cell expressed developmentally down‐regulated protein (NEDD4)”, and “dystrophin”, are essential for successful fertilization via calcium oscillations. These results more strongly suggested that PAWP is a sperm factor like PLCζ.

## 6 THE STRUCTURE AND FUNCTION OF PAWP

The molecular cloning of PAWP has been reported in human, mouse, and cow (Wu et al., 2007). These clones consisted of 309 (human), 354 (mouse), and 313 amino acids (cow). The N‐terminal half of PAWP shares 49% sequence similarity with WBP2. Wu et al. (2007) showed that the C‐terminal half of PAWP contains an unidentified repeating Tyr‐Gly‐X‐Pro‐Pro‐*X*‐Gly (YG*X*PP*X*G where *X* represents any residue) motif. Two (the number is species‐variable) PY motifs representing a consensus binding motif for WWI domain are also present in this region of PAWP (Figure 2b).

Although it has been shown that an injection of PAWP protein or cRNA can induce calcium oscillations, the detailed mechanism of how PAWP can induce calcium oscillations during oocyte activation remains unclear. It has been hypothesized that PAWP is involved in induction of oocyte activation via interaction(s) with other protein(s), e.g., yes‐associated protein (YAP) which has been reported to be involved in the interaction with PAWP (Wu et al., 2007). YAP is highly expressed in oocytes and possesses an SH3 binding motif (Chen & Sudol, 1995), suggesting the possibility that PLC becomes noncanonically activated through its SH3 domain to cleave PI(4,5)P_2_, as is the case in vertebrate fertilization.

The information about species differences in PAWP is very limited, but an injection of human PAWP can induce oocyte activation in Xenopus (Aarabi et al., 2010) and mouse and human (Aarabi et al., 2014b). Since there are some different nucleotides among the species, PAWP derived from different species may possess different activities, as PLCζ does.

## 7 FUTURE PROSPECTIVE SPERM FACTOR RESEARCH

After the discovery of PLCζ, several independent research groups separately published papers that confirmed the important role of PLCζ as a sperm factor (Table 1), but PAWP, the more recently identified candidate sperm factor, remains to be clarified. One of the major concerns is that only one group, Oko and his colleagues (Aarabi et al., 2014a, 2014b; Wu et al., 2007) confirmed the phenomenon caused by PAWP as a sperm factor. Another group attempted to reproduce the results reported by Dr. Oko and his colleagues (Aarabi et al., 2014a, 2014b; Wu et al., 2007). but failed to induce calcium oscillation with an injection of PAWP injection, at least in mouse(Nomikos, Sanders, et al., 2014) and human (Nomikos et al., 2015). In the latter study, the authors also did not observe the blockage of calcium oscillations by the injection of a competitive (PPGY) peptide for PAWP. The same research group was unable to hydrolyze PI(4,5)P_2_ in vitro and activate or modulate PLC activity (Nomikos, Theodoridou, et al., 2014).

However, several studies demonstrated that PAWP expression is correlated with fertility in pig (Sutovsky, 2015), cow (Kennedy et al., 2014), and human (Aarabi et al., 2014b; Sutovsky, Aarabi, Miranda‐Vizuete, & Oko, 2015), suggesting that PAWP may have other roles in the process of fertilization. Although we still do not know what causes opposite results from different research groups, further studies focusing on the underlying molecular mechanisms can be expected to clarify PAWP function.

Another concern is that only human PAWP protein or cRNA was used in the previous studies to examine the ability to induce calcium oscillations, although the successful molecular cloning of PAWP was reported, at least in mouse, human, and cow (Table 1, Wu et al., 2007). A similar mechanism of oocyte activation may well be shared among mammals. If PAWP can work as a true sperm factor in not only human but also other mammalian species, the molecules should be widely conserved among the species.

To resolve the controversy regarding PLCζ and PAWP, mouse that were deficient in PLCζ (Hachem et al., 2017; Nozawa, Satouh, Fujimoto, Oji, & Ikawa, 2018) or PAWP (Satouh, Nozawa, & Ikawa, 2015) were generated and their fertility was analyzed. Hachem et al. (2017) demonstrated that the PLCζ‐null sperm failed to trigger calcium oscillations, but some oocytes penetrated by PLCζ‐null sperm activated and developed into offspring. Nozawa et al. (2018) also showed that PLCζ‐null male mouse sired healthy pups consistently, although the number of pups per copulation was significantly reduced. These results strongly suggest that oocyte activation may eventually occur via alternative pathways if PLCζ is deficient.

In contrast, sperm from PAWP‐deficient mouse induced oocyte activation with calcium oscillations and produced pups with the same efficiency as sperm from wild‐type mouse (Satouh et al., 2015). It was recently suggested that there is a complementary factor of PAWP such as WBP2 (Hamilton et al., 2018). WBP2 is known as an ortholog of PAWP; both have a PY motif associated with the induction of oocyte activation. Expression of WBP2 has been confirmed in sperm and testis of mouse, but in human and cows it is only expressed in testis. Recombinant WBP2 injection into mouse oocytes induced oocyte activation with efficiency that was similar to that of recombinant PAWP (Hamilton et al., 2018). These experimental findings from experiments using knockout models raised the possibility that there is another oocyte activation pathway other than PLCζ and PAWP.

It has been recognized that citrate synthase (CS) becomes to be another candidate as a sperm factor in nonmammalian species. Harada et al. (2007) purified from newt sperm a 45 kDa protein that induced oocyte activation accompanied by an intracellular Ca^2+^ increase when injected into unfertilized newt oocytes. The partial amino acid sequences of this 45 kDa protein were homologous to X.laevis CS. (Harada et al., 2007). They also showed that injection of porcine CS induced a Ca^2+^ increase in newt oocyte and caused oocyte activation (Harada et al., 2007). Interestingly, quail sperm also contain CS which is required for long‐lasting, spiral‐like Ca^2+^ oscillations within the activated oocyte, which is essential for cell cycle progression in early embryos (Mizushima et al., 2014). These findings suggest that CS is now a strong candidate for a sperm factor which is conserved at least in nonmammalian species. Very recently, development of CS knockout mouse has been reported (Kang et al., in abstract). The CS knockout mice are subfertility with as small litters as PLCz KO mouse. Although further experiments and the publication of the data are required, it seems that CS has more essential role in oocyte activation than PAWP in mammals.

Although the controversy regarding the mechanisms underlying fertilization has focused on calcium ion, it is believed that zinc signaling also has an important role in oocyte activation. Que et al. (2015) reported that the fertilization of a mature and zinc‐enriched mouse oocyte triggered the ejection of zinc into the extracellular milieu in a series of coordinated events that they termed the 'zinc spark'. A zinc spark was also observed in human and cow oocytes following fertilization and oocyte activation induced by injection of PLCζ (Duncan et al., 2016; Que et al., 2019). These reports established that the zinc spark is a highly conserved event, at least in several mammalian species.

It has also been shown that meiotic resumption can be triggered artificially with the use of TPEN, a chelator of zinc, and the meiotic resumption can be inhibited by overloading the mouse oocyte with zinc ionophores (Bernhardt, Kong, Kim, O'Halloran, & Woodruff, 2012; Suzuki, Yoshida, Suzuki, Okuda, & Perry, 2010). In mouse, TPEN treatment was shown to be sufficient to activate metaphase II‐arrested oocytes injected with 'inactivated' sperm heads in the absence of intracellular calcium oscillations, resulting in live births after embryo transfer (Suzuki et al., 2010). Thus, full‐term development seems not to be dependent on calcium release during metaphase II exit, and calcium oscillations do not assure an altered rate of full‐term development compared to a single calcium rise.

The significance of the zinc spark is still unclear. Zinc ions must accumulate in the oocyte in order to spark at fertilization. Kim, Vogt, O'Halloran, and Woodruff (2010) showed that (a) zinc sparks could not be induced in immature oocytes that exist in the mouse ovary, and (b) the acute accumulation of zinc during meiotic maturation is important. Zinc spark profiles revealed that zygotes that developed into blastocysts released more zinc than those that failed to develop, and the rate of embryo development and the total cell number were higher than those of blastocysts (Zhang, Duncan, Que, O'Halloran, & Woodruff, 2016). It has thus been speculated that in mouse models, the amount of zinc ions at the zinc sparks can serve as an early biomarker of zygote quality. Based on the above‐described findings, zinc signaling also should be part of discussions about which molecule(s) are a true sperm factor. We believe that PLC zeta plays a major role in oocyte activation as sperm factor, but oocyte activation is not necessarily due to PLC zeta alone. Future research designs should thus consider that induction of oocyte activation may be due to multiple factors and pathways.
